# Associations of Serum Vitamin D With Dental Caries and Periodontitis: The HUNT Study

**DOI:** 10.1016/j.identj.2024.03.005

**Published:** 2024-04-01

**Authors:** Ernest Obeng Asante, Yue Chen, Rannveig Sakshaug Eldholm, Hedda Høvik, Marit Kolberg, Håvard Kjesbu Skjellegrind, Roya Torabi-Gaarden, Xiao-Mei Mai, Yi-Qian Sun

**Affiliations:** a Center for Oral Health Services and Research Mid-Norway (TkMidt), Trondheim, Norway; b Department of Clinical and Molecular Medicine, NTNU, Norwegian University of Science and Technology, Trondheim, Norway; c School of Epidemiology and Public Health, Faculty of Medicine, University of Ottawa, Ottawa, Ontario, Canada; d Department of Neuromedicine and Movement Science, NTNU, Norwegian University of Science and Technology, Trondheim, Norway; e Department of Geriatrics, Clinic of Medicine, St. Olavs Hospital, Trondheim, Norway; f HUNT Research Centre, Department of Public Health and Nursing, NTNU, Norwegian University of Science and Technology, Levanger, Norway; g Levanger Hospital, Nord-Trøndelag Hospital Trust, Levanger, Norway; h Department of Public Health and Nursing, NTNU, Norwegian University of Science and Technology, Trondheim, Norway; i Department of Pathology, Clinic of Laboratory Medicine, St. Olavs Hospital, Trondheim, Norway

**Keywords:** 25(OH)D, Dental caries, Periodontitis, HUNT study

## Abstract

**Objective:**

To study the relationships of serum 25-hydroxyvitamin D [25(OH)D] with dental caries and periodontitis in a general Norwegian adult population.

**Methods:**

We analysed a subsample of 1605 participants from the Trøndelag Health Study (HUNT) in Norway that had serum 25(OH)D levels measured in HUNT3 (2006-08) and oral health assessed in the HUNT4 Oral Health Study (2017-19). Negative binomial and Poisson regression models were used to estimate the ratios of means (RMs; for count oral outcomes) and prevalence ratios (PRs; for dichotomous oral outcomes).

**Results:**

Serum 25(OH)D was inversely associated with the number of decayed teeth in a dose-response gradient (<30.0 nmol/L: RM 1.41, 95% CI 1.07-1.85; 30.0-49.9 nmol/L: 1.14, 0.98-1.32 and ≥75.0 nmol/L: 0.84, 0.67-1.04, as compared to the 50.0-74.9 nmol/L group, *P* for trend <.001). Each 25 nmol/L decrease in 25(OH)D level was associated with a 15% (RM 1.15, 95% CI 1.05-1.26) increase in the mean number of decayed teeth. Serum 25(OH)D <30.0 nmol/L was associated with a 35% higher prevalence of severe periodontitis (PR 1.35, 95% CI 1.00-1.83). No association was observed between 25(OH)D and the number of natural teeth.

**Conclusion:**

The present study suggested that serum 25(OH)D level had an inverse and dose-response association with the number of decayed teeth, and serum 25(OH)D <30 nmol/L was associated with a higher prevalence of severe periodontitis in this Norwegian adult population.

## Introduction

Dental caries and periodontitis are the most prevalent oral diseases worldwide.^1^ It was estimated that 2.3 billion persons had untreated caries in permanent teeth, and 796 million had severe periodontitis in 2017.^2^ Recent data from Norway suggested that untreated caries were common in adults of all age groups despite a reduction in the decayed missing and filled teeth (DMFT) index over the past 45 years.^3^ Additionally, 17.6% of this Norwegian adult population had severe periodontitis.^4^ Dental caries is characterized by acid-producing bacteria in the presence of fermentable carbohydrates that cause damage to the hard tooth structure.^5^ Periodontitis is a polymicrobial disease distinguished by inflammation of the periodontium and progressive destruction of the tissues that support the tooth.^6^^,^^7^ Both oral diseases are major causes of tooth loss, reducing quality of life and imposing a significant economic and public health burden.^2^^,^^8^^,^^9^

Vitamin D may be associated with dental caries and periodontitis due to the complex and multifactorial nature of these oral diseases.^10^ Vitamin D is crucial for calcium-phosphate metabolism and hard tissue mineralization, including the alveolar bone and teeth.^10^ It also has anti-inflammatory and immunomodulatory effects, including regulating innate and adaptive immunity that impacts the pathogenesis of oral diseases.^10^^,^^11^ These potential effects have prompted studies on the relationships of serum 25-hydroxyvitamin D [25(OH)D] with dental caries and periodontitis.^12^^,^^13^ Nevertheless, the current evidence is inconclusive, which can be attributed to discrepancies in study design, study population selection and oral disease diagnosis.^12^, ^13^, ^14^, ^15^, ^16^ In contrast to the extensive studies on 25(OH)D and dental caries in children, there is a significant knowledge gap in studies on the adult population,^12^^,^^16^ even if caries is common across all age groups.^2^^,^^3^ Comparing studies between children and adults may not be directly applicable, which warrants more studies in the adult population. The association between 25(OH)D and periodontitis remains uncertain despite reported inverse associations in some studies.^15^^,^^17^ Periodontal outcomes and case definitions for periodontitis varied across most previous studies, likely contributing to some inconsistent findings.^13^^,^^14^^,^^18^ Moreover, most study results cannot be generalized as they focused on specific population groups.^13^

Therefore, we aimed to study the relationships of serum 25(OH)D with dental caries and periodontitis in a general Norwegian adult population. We used the 2017 American Academy of Periodontology and the European Federation of Periodontology (2017 AAP/EFP) classification system for periodontitis diagnosis^19^ and considered a large panel of confounders. This classification system represents the most recent international consensus on diagnostic criteria. Furthermore, we examined whether the relationship between 25(OH)D and periodontitis differed among younger and older adult groups in which the prevalence of periodontitis was different.^4^

## Methods

### Study population

The Trøndelag Health Study (HUNT) has so far had 4 surveys, HUNT1 (1984-1986), HUNT2 (1995-1997), HUNT3 (2006-2008), and HUNT4 (2017-2019).^20^ The HUNT Study is one of the most extensive population-based cohort studies in Norway, targeting all residents aged 20 years or older living in the Nord-Trøndelag region of Norway. Each of the 4 surveys included a range of health and lifestyle-related questionnaires and clinical examinations. The HUNT4 Oral Health Study invited a 20% randomly selected sample (n = 7347) of the HUNT4 participants living in 6 larger municipalities. In total, 4933 (67%) attended an oral examination at field stations.^3^^,^^4^

In the current study, due to budget constraints for measuring serum 25(OH)D, a 60% random sample of the 4933 participants was selected (n = 2959). Among them, 1703 were also HUNT3 participants, and we measured their 25(OH)D levels in serum collected in HUNT3 (regarded as baseline). We excluded participants without information on oral variables in HUNT4 (n = 98). A total of 1605 individuals had complete data on serum 25(OH)D in HUNT3 and oral outcomes in HUNT4, representing our analysis data set. The selection of the study population is presented in Figure.

**Figure:**
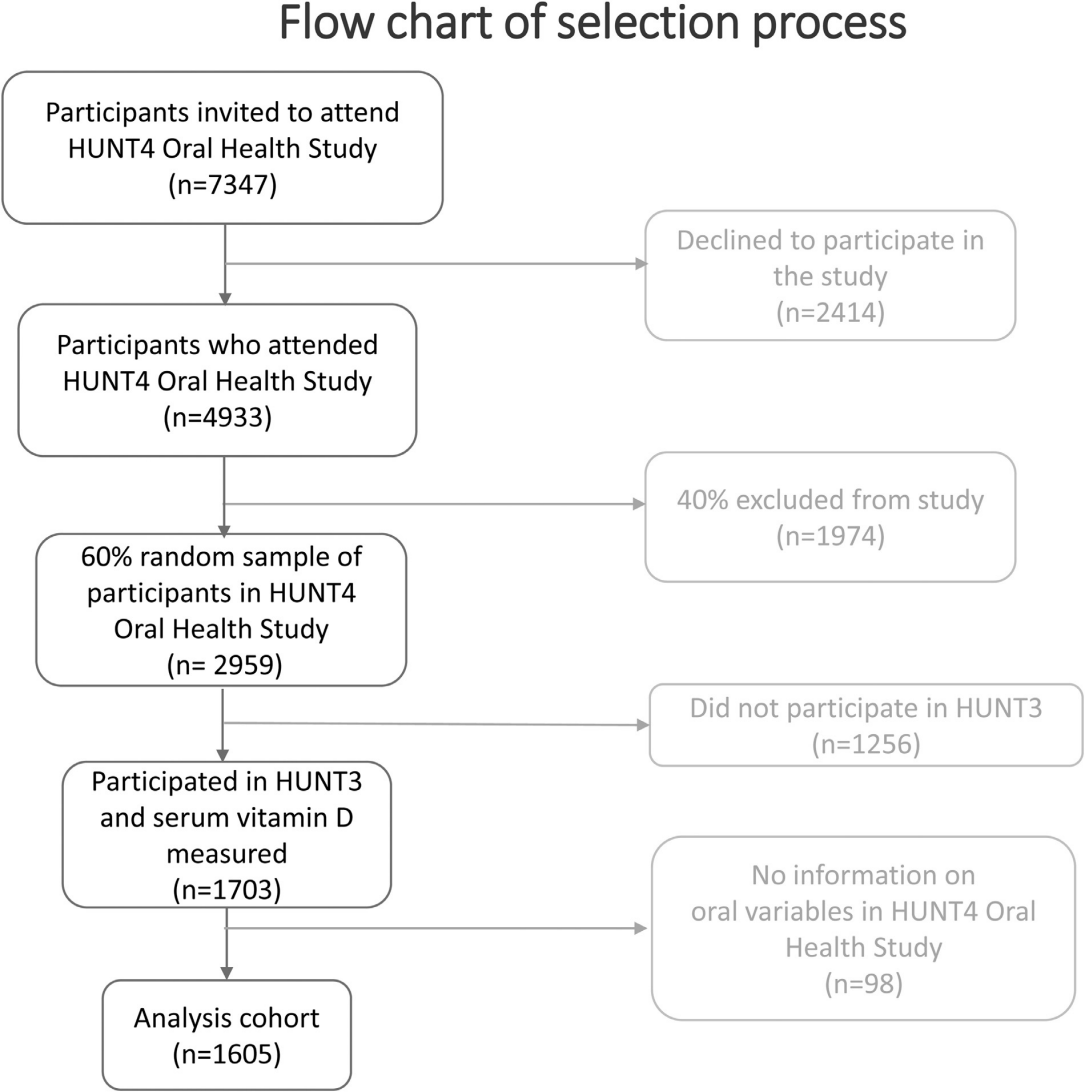
Flow chart of selection criteria for the analysis cohort of 1605 participants with information on serum 25(OH)D in HUNT3 and oral variables in the HUNT4 Oral Health Study. HUNT, Trøndelag Health Study.

### Serum 25(OH)D measurement

Collected blood samples in HUNT3 were stored at −80°C. Serum 25(OH)D was measured using LIAISON 25-OH vitamin D TOTAL (DiaSorin), an automated antibody-based chemiluminescence assay with a detection range of 10 to 375 nmol/L. We used a cosinor model based on the month of blood draw to estimate the seasonal-standardized 25(OH)D level (nmol/L), which represented the annual mean value of 25(OH)D for each participant.^21^^,^^22^ This takes into account the seasonal fluctuation in 25(OH)D level due to the geographical positioning of Trøndelag, Norway (Latitude 63.5° N). We treated seasonal-standardized 25(OH)D levels as a categorical variable with 4 categories (<30.0, 30.0-49.9, 50.0-74.9, and ≥75.0 nmol/L) and as a continuous variable (per 25 nmol/L decrease in serum 25(OH)D).^23^^,^^24^^,^^25^

### Oral health examination

Participants’ oral health status was evaluated by trained dentists or dental hygienists in the HUNT4 Oral Health Study. The evaluation comprised clinical and radiographic examination consisting of bitewing (BW) and orthopantomogram (OPG) radiographs. Dental caries and periodontitis, among other oral health outcomes, were assessed in participants. Detailed procedure and reliability of the assessments for dental caries and periodontitis in the HUNT4 Oral Health Study have been described elsewhere.^3^^,^^4^ A summarized version is presented below.

The number of decayed teeth was calculated using clinical and BW radiographic caries registration; third molars and persistent primary teeth were not included.^3^ Caries registrations were recorded using a 5-grade scale, where <3 was associated with enamel caries, and ≥3 comprised caries with dentine involvement.^26^ The D (decayed) teeth included caries lesions confined in dentine (grades 3-5), root remnants, secondary caries involving dentine and root caries with cavitation. The cumulative dentine caries experience DMFT, and the number of sound teeth were used for supplementary analysis. The M (missing) teeth were registered as missing regardless of the reason. The F (filled) teeth consisted of restorations without secondary caries and included all filling materials and crowns. Sound teeth were characterized by the absence of initial or dentine caries, filling and/or other restorations and did not include bridge abutment teeth.

The clinical periodontal examination involved periodontal probing depths and bleeding/suppuration on probing registered at 6 surfaces per tooth and tooth mobility grades 2 and 3.^4^ Using BW radiographs, a periodontitis case was determined by the distance between the cementum enamel junction and the alveolar bone crest exceeding 1.5 mm at ≥2 nonadjacent teeth. OPGs were used when BW examination was not performed.^4^ Confirmed cases were classified by stage and grade. Three periodontists performed radiographic examinations and periodontitis case classification. Based on the 2017 AAP/EFP classification system,^19^ periodontitis stage (1-4) was defined by radiographic alveolar bone loss, tooth loss due to periodontitis and complexity factors. All complexity factors of the 2017 classification system were considered.^4^^,^^19^ In the absence of clinical parameters, the staging was determined solely by radiographic evaluation, OPG and BW. Staging in periodontitis cases measures disease severity.^19^ The definition of periodontitis was influenced by age-related cumulative bone loss^27^ and a higher prevalence of lower stages of the disease in older populations.^28^^,^^29^ Thus, for the current study, participants with stage 3 or 4 periodontitis were defined as having severe periodontitis. Grading was defined by the percentage of bone loss relative to the participant's age.^4^ Grading is a measure of disease progression^19^ and was used in the supplementary analysis.

General dental health status was represented by the number of natural teeth, which included permanent teeth with the exception of third molars and persistent primary teeth.

### Covariates

Potential confounding variables were collected through questionnaires and clinical examination in HUNT3 (at baseline). Sociodemographic factors included age (as a continuous variable), sex, occupation (defined by Erikson Goldthorpe Portocarero social class scheme ranging from high to low social status: EGP class I-VII)^30^ and marital status [no (unmarried, widow/widower, divorced or separated), yes (married or registered partner)]. Body mass index (BMI) was calculated as weight divided by squared height and classified into underweight or normal (<25.0 kg/m²) (only 3 participants with BMI < 18.5 kg/m²), overweight (25.0-29.9 kg/m²) or obesity (≥30.0 kg/m²). Lifestyle factors included smoking status in packyears (pyrs) [never smokers, former smokers (0-10, 10.1-20, or >20 pyrs), current smokers (0-10, 10.1-20, or >20 pyrs)], alcohol consumption per month (never, 1-4 or ≥5 times) and physical activity levels (inactive, low, moderate, or high).^31^ Diabetes (no, yes) among participants was determined by the question “Have you had or do you have diabetes?” and/or had a non-fasting blood glucose level above 11 mmol/L. Additional health, dietary and lifestyle-related covariates included osteoporosis (no, yes) based on the question “Have you had or do you have osteoporosis?,” milk and sugary soft drink intake (rarely or never, 1-6 glass per week or one or more glass per day) and smokeless tobacco use (no, yes). Symptoms of depression were collected using the Hospital Anxiety and Depression Scale (HADS) and categorized as 0 to 7 or ≥8 (depressive symptoms).^32^ Missing information on all covariates was treated as an “unknown” category for each variable and was included in the statistical analyses.

### Statistical analyses

The relationships of serum 25(OH)D level with the number of decayed teeth and the number of natural teeth were evaluated using negative binomial regression models to estimate the ratios of means (RMs) with 95% confidence intervals (CIs), which accounts for count dependent variables with potential overdispersion.^33^ Poison regression with robust error variance was used to estimate prevalence ratios (PRs) for the relationship between serum 25(OH)D and severe periodontitis.^34^ RMs were also estimated for the relationships of 25(OH)D with DMFT, the number of sound teeth and periodontal grades in supplementary analyses.

We used multivariable analysis to control for potential confounders, including age, sex, BMI, occupation, marital status, smoking status in packyears, alcohol consumption, physical activity and diabetes in Model 1 (main model). Model 2 additionally adjusted for osteoporosis, milk consumption, sugary soft drink intake, smokeless tobacco use and depressive symptoms. Potential confounders were included based on literature regarding serum 25(OH)D with caries and periodontitis.^15^^,^^35^^,^^36^ Effect modification by age (<60 or ≥60 years in HUNT4) for the association between 25(OH)D and periodontitis was evaluated by a likelihood ratio test.

Analyses were conducted using STATA/MP Version 17 (StataCorp LP).

## Results

The mean age of the study population at baseline (HUNT3) was 49.0 years. Participants with 25(OH)D levels of <30.0 and 30.0–49.9 nmol/L were younger, with a lower representation of females and a higher likelihood of obesity than those with serum 25(OH)D levels of 50.0 nmol/L or above. In addition, participants in the 25(OH)D <30.0 nmol/L group were less likely to be from the highest social class, be married or be physically active, but they were more likely to smoke, use smokeless tobacco or have depressive symptoms (Table 1).

**Table 1:** Baseline characteristics of participants in the HUNT3 survey stratified by seasonal-standardized serum 25(OH)D level.

	Seasonal-standardized serum 25(OH)D level (nmol/L)				
Characteristics	<30.0 n = 94	30.0-49.9 n = 590	50.0-74.9 n = 714	≥75 n = 207	Total n = 1605
**Age (years)**	45.6 ± 14.1	47.8 ± 12.6	50.3 ± 12.5	49.6 ± 13.4	49.0 ± 12.8
**Sex**					
Female	48 (51.1)	322 (54.6)	429 (60.1)	137 (66.2)	936 (58.3)
Male	46 (48.9)	268 (45.4)	285 (39.9)	70 (33.8)	669 (41.7)
**Body mass index (kg/m^2^)**					
Underweight or normal (<25.0)	21 (22.3)	168 (28.5)	281 (39.4)	98 (47.3)	568 (35.4)
Overweight (25.0-29.9)	41 (43.6)	251 (42.5)	329 (46.1)	88 (42.5)	709 (44.2)
Obesity (≥30.0)	32 (34.1)	169 (28.6)	104 (14.6)	21 (10.1)	326 (20.3)
Unknown	0 (0)	2 (0.3)	0 (0)	0 (0)	2 (0.1)
Occupation*					
EGP Class I	9 (9.6)	109 (18.5)	152 (21.3)	48 (23.2)	318 (19.8)
EGP Class II	20 (21.3)	125 (21.2)	182 (25.5)	53 (25.6)	380 (23.7)
EGP Class III	31 (33.0)	197 (33.4)	207 (29.0)	57 (27.5)	492 (30.7)
EGP Class IV	4 (4.1)	39 (6.6)	36 (5.0)	13 (6.3)	92 (5.7)
EGP Class V + VI	18 (19.1)	93 (15.8)	91 (12.7)	24 (11.6)	226 (14.1)
EGP Class VII	6 (6.4)	16 (2.7)	28 (3.9)	7 (3.4)	57 (3.6)
Unknown	6 (6.4)	11 (1.9)	18 (2.5)	5 (2.4)	40 (2.5)
**Marital status**					
No†	54 (57.4)	235 (39.8)	237 (33.2)	82 (39.6)	608 (37.9)
Yes‡	40 (42.6)	355 (60.2)	476 (66.7)	125 (60.4)	996 (62.1)
Unknown	0 (0)	0 (0)	1 (0.1)	0 (0)	1 (0.1)
**Smoking status in packyears (pyrs)**					
Never smokers	37 (39.4)	275 (46.6)	340 (47.6)	95 (45.9)	747 (46.5)
Former smokers 0-10 pyrs	14 (14.9)	108 (18.3)	124 (17.4)	40 (19.3)	286 (17.8)
Former 10.1-20 pyrs	8 (8.5)	37 (6.3)	48 (6.7)	16 (7.7)	109 (6.8)
Former >20 pyrs	3 (3.2)	22 (3.7)	29 (4.1)	7 (3.4)	61 (3.8)
Current smokers 0-10 pyrs	14 (14.9)	43 (7.3)	48 (6.7)	9 (4.3)	114 (7.1)
Current 10.1-20 pyrs	6 (6.4)	28 (4.7)	30 (4.2)	13 (6.3)	77 (4.8)
Current >20 pyrs	5 (5.3)	26 (4.4)	19 (2.7)	3 (1.4)	53 (3.3)
Unknown	7 (7.4)	51 (8.6)	76 (10.6)	24 (11.6)	158 (9.8)
**Alcohol consumption**					
Never	9 (9.6)	16 (2.7)	12 (1.7)	4 (1.9)	41 (2.6)
1-4 times per month	71 (75.5)	487 (82.5)	547 (76.6)	157 (75.8)	1262 (78.6)
≥5 times per month	14 (14.9)	83 (14.1)	146 (20.4)	45 (21.7)	288 (17.9)
Unknown	0 (0)	4 (0.7)	9 (1.3)	1 (0.5)	14 (0.9)
**Physical activity**					
Inactive	6 (6.4)	46 (7.8)	50 (7.0)	4 (1.9)	106 (6.6)
Low	19 (20.2)	88 (14.9)	81 (11.3)	19 (9.2)	207 (12.9)
Moderate	22 (23.4)	170 (28.8)	211 (29.6)	76 (36.7)	479 (29.8)
High	6 (6.4)	73 (12.4)	105 (14.7)	46 (22.2)	230 (14.3)
Unknown	41 (43.6)	213 (36.1)	267 (37.4)	62 (30.0)	583 (36.3)
**Diabetes**					
No	91 (96.8)	557 (94.4)	684 (95.8)	192 (92.8)	1524 (95.0)
Yes	3 (3.2)	20 (3.4)	18 (2.5)	9 (4.3)	50 (3.1)
Unknown	0	13 (2.2)	12 (1.7)	6 (2.9)	31 (1.9)
**Osteoporosis**					
No	87 (92.6)	581 (98.5)	698 (97.8)	198 (95.7)	1564 (97.5)
Yes	3 (3.2)	3 (0.5)	12 (1.7)	5 (2.4)	23 (1.4)
Unknown	4 (4.3)	6 (1.0)	4 (0.6)	4 (1.9)	18 (1.1)
**Milk intake**					
Rarely or never	39 (41.5)	186 (31.5)	222 (31.1)	59 (28.5)	506 (31.5)
1-6 glass per week	21 (22.3)	158 (26.8)	164 (23.0)	44 (21.3)	387 (24.1)
1 or more glass per day	31 (33.0)	229 (38.8)	309 (43.3)	97 (46.9)	666 (41.5)
Unknown	3 (3.2)	17 (2.9)	19 (2.7)	7 (3.4)	46 (2.9)
**Sugary soft drink intake**					
Rarely or never	40 (42.6)	304 (51.5)	398 (55.7)	122 (58.9)	864 (53.8)
1-6 glass per week	41 (43.6)	204 (34.6)	230 (32.2)	60 (29.0)	535 (33.3)
1 or more glass per day	11 (11.7)	63 (10.7)	58 (8.1)	17 (8.2)	149 (9.3)
Unknown	2 (2.1)	19 (3.2)	28 (3.9)	8 (3.9)	57 (3.6)
**Smokeless tobacco use**					
No	73 (77.7)	485 (82.2)	627 (87.8)	179 (86.5)	1364 (85.0)
Yes	20 (21.3)	94 (15.9)	76 (10.6)	25 (12.1)	215 (13.4)
Unknown	1 (1.1)	11 (1.9)	11 (1.5)	3 (1.5)	26 (1.6)
**Depressive symptoms (HADS)**					
0-7	64 (68.1)	460 (78.0)	559 (78.3)	170 (82.1)	1253 (78.1)
≥8	12 (12.8)	34 (5.8)	46 (6.4)	10 (4.8)	102 (6.4)
Unknown	18 (19.2)	96 (16.3)	109 (15.3)	27 (13.0)	250 (15.6)

Data are given as the number of participants (column percentage) or mean ± SD.

25(OH)D, 25-hydroxyvitamin D; EGP, Erikson Goldthorpe Portocarero social class scheme; HADS, Hospital Anxiety and Depressive symptoms Scale; HUNT, Trøndelag Health Study.

⁎ Occupation: EGP Class I (administrative managers, politicians or academic professions), EGP Class II (occupations with shorter college and university degrees), EGP Class III (office and customer service occupations, sales, service and care professions), EGP Class IV (occupations in agriculture, forestry and fishing), EGP Class V + VI (craftsmen, process and machine operators or transport), EGP Class VII (occupations without education requirements).

† No: Unmarried, widow/widower, divorced or separated.

‡ Yes: married or registered partner.

The mean number of decayed teeth was 1.3 (range 0-16). In the crude model (Table 2), serum 25(OH)D level was inversely associated with the number of decayed teeth (<30.0 nmol/L: RM 1.50, 95% CI 1.14-1.97; 30.0-49.9 nmol/L: 1.17, 1.01-1.35 and ≥75.0 nmol/L: 0.79, 0.63-0.98, as compared to the 50.0-74.9 nmol/L group, *P* for trend <.001). The association attenuated to some degree after adjustments for confounders in Model 1 (<30.0 nmol/L: RM 1.41, 95% CI 1.07-1.85; 30.0-49.9 nmol/L: 1.14, 0.98-1.32 and ≥75.0 nmol/L: 0.84, 0.67-1.04, *P* for trend <.001). Additional adjustments for potential confounders in Model 2 provided similar estimates. Each 25 nmol/L decrease in serum 25(OH)D was associated with a 15% increase in the mean number of decayed teeth (RM 1.15, 95% CI 1.05-1.26). Supplementary analyses (Supplementary Tables 2 and 3, Model 1) showed weak associations in both the lowest and highest 25(OH)D categories for the DMFT and the number of sound teeth.

**Table 2:** The association between seasonal-standardized serum 25(OH)D level and the number of decayed teeth (n = 1605).

Serum 25(OH)D (nmol/L)	n	Number of decayed teeth	Ratio of means (95% CI)		
		Mean (range)	Crude model	Model 1*	Model 2†
Categorical					
<30.0	94	1.9 (0-16)	1.50 (1.14-1.97)	1.41 (1.07-1.85)	1.46 (1.11-1.93)
30.0-49.9	590	1.5 (0-13)	1.17 (1.01-1.35)	1.14 (0.98-1.32)	1.14 (0.99-1.32)
50.0-74.9	714	1.3 (0-13)	1.00 (reference)	1.00 (reference)	1.00 (reference)
≥75.0	207	1.0 (0-7)	0.79 (0.63-0.98)	0.84 (0.67-1.04)	0.84 (0.68-1.05)
P for trend			<0.001	<0.001	<0.001
Continuous‡	1605	1.3 (0-16)	1.20 (1.10-1.31)	1.15 (1.05-1.26)	1.16 (1.06-1.27)

25(OH)D, 25-hydroxyvitamin D; 95% CI, 95% confidence interval; n, number of participants.

⁎ Model 1 adjusted for age, sex, body mass index, occupation, marital status, smoking status in packyears, alcohol consumption, physical activity and diabetes.

† Model 2 adjusted for osteoporosis, milk intake, sugary soft drink intake, smokeless tobacco use and depressive symptoms in addition to model 1.

‡ Per 25 nmol/L decrease in serum 25(OH)D.

Severe periodontitis was observed in 375 (23%) out of 1605 participants (Table 3). Serum 25(OH)D level of <30.0 nmol/L was associated with a 35% higher severe periodontitis prevalence in Model 1 (<30.0 nmol/L: PR 1.35, 95% CI 1.00-1.83; 30.0-49.9 nmol/L: 0.91, 0.75-1.09 and ≥75.0 nmol/L: 0.95, 0.74-1.23, as compared to the 50.0-74.9 nmol/L group). No association was observed when 25(OH)D was used as a continuous variable (PR 1.03, 95% CI 0.92-1.15 per 25 nmol/L decrease). Additional analyses showed no associations with periodontal grades or the number of natural teeth (Supplementary Table 4 and Table 4).

**Table 3:** The association between seasonal-standardized serum 25(OH)D level and prevalence of severe periodontitis (n = 1605).

Serum 25(OH)D (nmol/L)	n	Cases	Prevalence (%)	Prevalence ratio (95% CI)		
				Crude model	Model 1*	Model 2†
Categorical						
<30.0	94	28	30	1.21 (0.89-1.70)	1.35 (1.00-1.83)	1.37 (1.00-1.87)
30.0-49.9	590	123	21	0.85 (0.69-1.04)	0.91 (0.75-1.09)	0.92 (0.77-1.11)
50.0-74.9	714	175	25	1.00 (reference)	1.00 (reference)	1.00 (reference)
≥75.0	207	49	24	0.97 (0.73-1.27)	0.95 (0.74-1.23)	0.93 (0.72-1.21)
*P* for trend				.80	.58	.41
Continuous‡	1605	375	23	0.98 (0.88-1.10)	1.03 (0.92-1.15)	1.05 (0.94-1.18)

25(OH)D, 25-hydroxyvitamin D; 95% CI, 95% confidence interval; n, number of participants.

⁎ Model 1 adjusted for age, sex, body mass index, occupation, marital status, smoking status in packyears, alcohol consumption, physical activity and diabetes.

† Model 2 adjusted for osteoporosis, milk intake, sugary soft drink intake, smokeless tobacco use and depressive symptoms in addition to model 1.

‡ Per 25 nmol/L decrease in serum 25(OH)D.

**Table 4:** The association between seasonal-standardized serum 25(OH)D level and the number of natural teeth (n = 1605).

Serum 25(OH)D (nmol/L)	n	Number of natural teeth	Ratio of means (95% CI)		
		Mean (range)	Crude model	Model 1*	Model 2†
Categorical					
<30.0	94	24.5 (2-28)	0.98 (0.94-1.02)	0.98 (0.93-1.02)	0.98 (0.93-1.02)
30.0-49.9	590	25.0 (5-28)	1.00 (0.98-1.02)	0.99 (0.97-1.02)	0.99 (0.97-1.01)
50.0-74.9	714	25.0 (4-28)	1.00 (reference)	1.00 (reference)	1.00 (reference)
≥75.0	207	24.7 (2-28)	0.99 (0.96-1.02)	0.98 (0.95-1.01)	0.98 (0.95-1.01)
P for trend			0.92	0.81	0.76
Continuous‡	1605	24.9 (2-28)	1.00 (0.99-1.01)	1.00 (0.98-1.01)	1.00 (0.98-1.01)

25(OH)D, 25-hydroxyvitamin D; 95% CI, 95% confidence interval; n, number of participants.

⁎ Model 1 adjusted for age, sex, body mass index, occupation, marital status, smoking status in packyears, alcohol consumption, physical activity and diabetes.

† Model 2 adjusted for osteoporosis, milk intake, sugary soft drink intake, smokeless tobacco use and depressive symptoms in addition to model 1.

‡ Per 25 nmol/L decrease in serum 25(OH)D.

The prevalence of severe periodontitis was 8.7% (n = 71) and 38.6% (n = 304) in participants <60 and ≥60 years in HUNT4, respectively. The likelihood ratio test showed no evidence of modification by age (<60 and ≥60 years in HUNT4) in the association between 25(OH)D and severe periodontitis (*P* = .79).

## Discussion

We found an inverse and dose-response association between serum 25(OH)D level and the number of decayed teeth. Each 25 nmol/L decrease in serum 25(OH)D level was associated with a 15% increase in the mean number of decayed teeth. Also, the prevalence of severe periodontitis was 35% higher in participants with serum 25(OH)D <30.0 nmol/L compared to the level of 50.0 to 74.9 nmol/L. No association was observed between serum 25(OH)D level and the number of natural teeth. Age did not modify the association between serum 25(OH)D level and periodontitis.

Similar to our findings, a cross-sectional study by Zhou et al^36^ has reported an inverse association between serum 25(OH)D and dental caries in adults in the United States. An increase of 10 nmol/L serum 25(OH)D was associated with a 7% decrease in caries, whereas the lowest 25(OH)D group of <25 nmol/L was 2.48 times more likely to be affected by caries than the reference group of ≥75 nmol/L.^36^ A meta-analysis of all age groups found a dose-response relationship, associating a 10 nmol/L increase in 25(OH)D level with a 3% decrease in caries prevalence.^12^ However, there was uncertainty in the evidence, likely because of the age range (1-80 years) and the current low number of studies in the adult population.^12^

In contrast to the limited number of studies in the adult population, the relationship between 25(OH)D and dental caries in children has been widely documented, with a recent meta-analysis reporting an inverse relationship.^16^ However, caries experience in children's studies has largely been associated with primary dentition,^35^ which requires a shorter exposure time. Primary and permanent dentition differ in morphology and histology, resulting in varying susceptibilities to demineralization.^37^ Additionally, some studies in children may have limited robustness and generalizability due to small sample sizes and a lack of adjustment of important confounding variables such as socioeconomic factors.^16^^,^^35^ These factors should be considered when comparing studies in children and adults.

Our results showed a slightly increased caries experience proxied by DMFT in the <30.0 nmol/L and ≥75.0 nmol/L 25(OH)D groups. The weak association between 25(OH)D <30.0 nmol/L and DMFT was consistent with our results for the number of decayed teeth. One possible explanation for a weak increase of DMFT in those with 25(OH)D ≥75.0 nmol/L is that DMFT is a cumulative measure and some of the participants with missing or filled teeth might have supplemented vitamin D before serum 25(OH)D was measured.

Consistent with our results, case-control studies have reported considerably lower 25(OH)D levels in patients with severe periodontitis stages compared to healthy controls,^15^^,^^17^ supported by population-based cross-sectional studies.^38^, ^39^, ^40^, ^41^ Clinical trials also suggested that vitamin D supplements improved overall periodontal health^42^; however, limited evidence exists from longitudinal studies.^13^^,^^14^ A Finnish case-control study reported no association between 25(OH)D and periodontal health status.^43^ Though the lack of association in the Finnish study can be attributed to the general low levels of 25(OH)D in the population,^44^ other variations in population characteristics, including genetic profile have been associated with such discrepancies.^45^^,^^46^

Moreover, as presented in reviews, there is significant heterogeneity in periodontal outcomes and periodontitis case definitions in literature.^13^, ^14^, ^15^^,^^42^ Numerous assessment methods, mostly based on previous periodontitis classification, have been utilized in studies. Differences in periodontitis case definition may also account for some study discrepancies.^14^^,^^18^^,^^47^ For instance, due to the reliance on radiographic alveolar bone loss for periodontitis assessment, age-related cumulative bone loss could lead to higher periodontitis prevalence in our older study population,^27^ potentially contributing to discrepancies with other studies. These discrepancies may be reduced with the 2017 AAP/EFP case definition incorporating clinical data for comprehensive disease diagnosis.^19^ Indeed, studies utilizing the 2017 AAP/EFP case definition or related consensus are mostly comparable due to high diagnostic accuracy for severe periodontitis.^48^ Such conformity regarding periodontitis diagnosis is necessary to clarify this association.

The number of natural teeth is a proxy measure for general oral health.^49^ Studies showed that the predominant factors for tooth loss were oral diseases, particularly caries and periodontitis.^9^^,^^50^ Contrary to our results, a cross-sectional study found an inverse association between serum 25(OH)D level and tooth loss in Koreans aged ≥50 years.^39^ However, the association was not significant in females after adjusting for confounders. A previous study reported that each 25 nmol/L increase in serum 25(OH)D level was associated with a 13% decreased risk of tooth loss in a 5-year follow-up.^51^ In this referred study, the protective effect of 25(OH)D on tooth loss seemed to be partially mediated by its effect on periodontitis.^51^ In a 3-year randomized control trial, calcium and vitamin D supplementation reduced the risk of tooth loss in older adults, but the independent effect of vitamin D could not be determined.^52^ In our study, some teeth might have been lost before HUNT3, and serum 25(OH)D might have had a minor impact on complicated cases of caries and periodontitis that led to tooth loss between HUNT3 and HUNT4; these factors may have contributed to the observed null association between serum 25(OH)D level and the number of natural teeth.

Vitamin D has a multifaceted effect on dental caries and periodontitis. The initial stage of caries formation is indicated by white spot lesions on the enamel, which suggests an early demineralization stage.^53^ Vitamin D regulates calcium-phosphate homeostasis, which is essential for the formation, calcification, mineralization and maintenance of the alveolar bone and teeth.^10^ Optimum vitamin D levels in saliva can support the remineralization of early enamel lesions, as demonstrated in an in vivo and in vitro study.^54^ However, a deficiency in vitamin D could affect salivary secretion and the potential teeth mineralization process.^55^ He et al. showed an increased salivary flow rate with a high concentration of antimicrobial peptides in response to a 14-week vitamin D3 supplementation.^56^ The role of vitamin D in inducing the production of antimicrobial peptides capable of reducing cariogenic bacteria has been reported.^57^^,^^58^ These antimicrobial peptides are an important part of innate immunity,^58^ that also defend against periodontal pathogens to reduce the accumulation of plaque and calculus.^59^ Generally, periodontitis is induced by an unbalanced interaction between periodontal pathogens and the host's inflammatory response.^6^ Vitamin D regulates immune response by suppressing the production of pro-inflammatory cytokines involved in the development of periodontitis.^7^ Furthermore, vitamin D's bone remodelling effect slows the progression of alveolar bone loss and the resulting tooth loss in periodontitis.^7^

The current study has the following strengths. The analysis data set was derived from a general population. Caries and periodontitis were defined based on detailed clinical and radiographic examinations. We used the standardized dental outcome definitions, including the 2017 AAP/EFP classification for periodontitis. Additionally, we adjusted for a large panel of confounders missing in previous studies.

This study also has limitations. First, we cannot exclude possible selection bias. Attrition from HUNT3 to HUNT4 was reported to be higher among older adults and individuals with chronic diseases or poor self-reported health.^20^ Oral health examinations were performed in field stations with a participation rate of 67%. Thus, older adults showing greater levels of frailty were more likely to decline an invitation to participate in the survey. Furthermore, participants excluded from the study (n = 3328) were younger (48.0 vs 59.4 years in HUNT4) than those in the analysis cohort (n = 1605) (Supplementary Table 1). The age difference was mainly explained by the fact that participants aged 20 to 30 years in HUNT4 did not participate in HUNT3 due to the age limit set at 20 in HUNT surveys. Apart from age, baseline characteristics were generally similar between the excluded and included participants. Second, a one-time 25(OH)D measurement may not reflect participants’ long-term vitamin D status. Third, we could not assess the incidence of caries and periodontitis from HUNT3 to HUNT4 in this retrospective cohort study as there was no information on oral outcomes in the HUNT3 Survey. Lastly, not all potential confounders were assessed in our analysis due to lack of information, such as diet or dietary habits.^60^ Nevertheless, we found that milk and sugary soft drink intake had no notable impact on the associations of interest.

## Conclusion

This study suggests an inverse and dose-response association between serum 25(OH)D level and the number of decayed teeth. Additionally, serum 25(OH)D <30 nmol/L was associated with a higher prevalence of severe periodontitis in this Norwegian adult population. The present findings highlight the potential role of vitamin D status in the development of caries and severe cases of periodontitis in adults. However, large prospective cohort studies with standardized dental outcome definitions are necessary to confirm these associations.

## Conflict of interest

None disclosed.

## Data Availability

Data from the HUNT Study used in research projects will, when reasonably requested by others, be made available on request to the HUNT Data Access Committee. The HUNT data access information describes the policy regarding data availability (https://www.ntnu.edu/hunt/data).

## Appendix Supplementary materials

Image, application 1

## Footnotes

Supplementary material associated with this article can be found in the online version at doi:10.1016/j.identj.2024.03.005.
