# Supplementary material for: Associations of Serum Vitamin D With Dental Caries and Periodontitis: The HUNT Study
Source: Int Dent J. 2024 Apr 1;74(3):500–9. doi: 10.1016/j.identj.2024.03.005 (PMC11123562; doi:10.1016/j.identj.2024.03.005)
Supplement: Supplementary file 1 [file mmc1.docx]

**Supplementary table 1**. HUNT4 characteristics of participants excluded from the study compared with those included in the analysis cohort.

|  |  | Excluded from study | Analysis cohort |
| --- | --- | --- | --- |
| Characteristics |  | n= 3328 | n= 1605 |
|  |  |  |  |
| Age (years) |  | 48.0 ± 16.9 | 59.4 ± 12.8 |
|  |  |  |  |
| Sex |  |  |  |
| Female |  | 1823 (54.8) | 936 (58.2) |
| Male |  | 1505 (45.2) | 669 (41.7) |
|  |  |  |  |
| Body mass index (kg/m^2^) |  |  |  |
| Underweight or normal (<25) |  | 1251 (37.6) | 493 (30.7) |
| Overweight (25.0–29.9) |  | 1308 (39.3) | 734 (45.7) |
| Obesity (≥30.0) |  | 736 (22.1) | 375 (23.4) |
| Unknown |  | 33 (1.0) | 3 (0.2) |
|  |  |  |  |
| Education |  |  |  |
| <=10 years |  | 215 (6.5) | 143 (8.9) |
| 11-13 years |  | 1570 (47.2) | 742 (46.2) |
| >=14 years |  | 1500 (45.1) | 717 (44.7) |
| Unknown |  | 43 (1.3) | 3 (0.2) |
|  |  |  |  |
| Marital status |  |  |  |
| No* |  | 1773 (53.3) | 585 (36.5) |
| Yes^¥^ |  | 1522 (45.7) | 1017 (63.4) |
| Unknown |  | 33 (1.0) | 3 (0.2) |
|  |  |  |  |
| Smoking status in packyears (pyrs) |  |  |  |
| Never smokers |  | 1513 (45.5) | 715 (44.6) |
| Former smokers 0–10 pyrs |  | 447 (13.4) | 254 (15.8) |
| Former 10.1–20 pyrs |  | 239 (7.2) | 158 (9.8) |
| Former >20 pyrs |  | 179 (5.4) | 104 (6.5) |
| Current smokers 0–10 pyrs |  | 51 (1.5) | 21 (1.3) |
| Current 10.1–20 pyrs |  | 86 (2.6) | 28 (1.7) |
| Current >20 pyrs |  | 92 (2.8) | 38 (2.4) |
| Unknown |  | 721 (21.7) | 287 (17.9) |
|  |  |  |  |
| Alcohol consumption |  |  |  |
| Never |  | 290 (8.7) | 127 (7.9) |
| 1–4 times per month |  | 2322 (69.8) | 1077 (67.1) |
| ≥ 5 times per month |  | 659 (19.8) | 386 (24.1) |
| Unknown |  | 57 (1.7) | 15 (1.0) |
|  |  |  |  |
| Physical activity^α^ |  |  |  |
| Never |  | 110 (3.3) | 33 (2.1) |
| Less often than once a week |  | 416 (12.5) | 170 (10.6) |
| Once a week |  | 594 (17.9) | 243 (15.1) |
| 2-3 times a week |  | 1402 (42.1) | 758 (47.2) |
| About every day |  | 747 (22.5) | 384 (23.9) |
| unknown |  | 59 (1.8) | 17 (1.1) |
|  |  |  |  |
| Diabetes |  |  |  |
| No |  | 3123 (93.8) | 1503 (93.6) |
| Yes |  | 136 (4.1) | 86 (5.4) |
| Unknown |  | 69 (2.1) | 16 (1.0) |

Data are given as the number of participants (column percentage) or mean ± SD

25(OH)D: 25-hydroxyvitamin D; EGP: Erikson Goldthorpe Portocarero social class scheme; HUNT: Trøndelag Health Study

*No: Unmarried, widow/widower, divorced or separated

^¥^Yes: married or registered partner

^α^ How often do you exercise?

**Supplementary table 2.** The association between seasonal-standardized serum 25(OH)D level and dental caries experience (DMFT) (n=1605)

| Serum 25(OH)D (nmol/L) |  | DMFT  Mean (range) | Ratio of means (95% CI) | | |
| --- | --- | --- | --- | --- | --- |
|  | n |  | Crude model | Model 1^a^ | Model 2^b^ |
| Categorical |  |  |  |  |  |
| <30.0 | 94 | 17.3 (1 – 28) | 1.00 (0.92 – 1.08) | 1.05 (0.99 – 1.11) | 1.05 (0.99 – 1.12) |
| 30.0–49.9 | 590 | 16.9 (0 – 28) | 0.97 (0.94 – 1.01) | 1.00 (0.97 – 1.03) | 1.00 (0.97 – 1.03) |
| 50.0–74.9 | 714 | 17.4 (0 – 28) | 1.00 (reference) | 1.00 (reference) | 1.00 (reference) |
| ≥75.0 | 207 | 17.8 (0 – 28) | 1.03 (0.97 – 1.09) | 1.05 (1.00 – 1.09) | 1.05 (1.01 – 1.09) |
| P for trend |  |  | 0.11 | 0.40 | 0.43 |
|  |  |  |  |  |  |
| Continuous^c^ | 1605 | 17.3 (0 – 28) | 0.98 (0.96– 1.01) | 0.99 (0.97 – 1.01) | 0.99 (0.97 – 1.01) |

25(OH)D: 25-hydroxyvitamin D; 95% CI: 95% confidence interval; DMFT: decayed, missing and filled teeth; n: number of participants

^a^ Model 1 adjusted for age, sex, body mass index, occupation, marital status, smoking status in packyears, alcohol consumption, physical activity and diabetes.

^b^ Model 2 adjusted for osteoporosis, milk intake, sugary soft drink intake, smokeless tobacco use and depressive symptoms in addition to model 1.

^c^ per 25 nmol/L decrease in serum 25(OH)D.

**Supplementary table 3.** The association between seasonal-standardized serum 25(OH)D level and the number of sound teeth (n=1605)

| Serum 25(OH)D (nmol/L) |  | Number of sound teeth  Mean (range) | Ratio of means (95% CI) | | |
| --- | --- | --- | --- | --- | --- |
|  | n |  | Crude model | Model 1^a^ | Model 2^b^ |
| Categorical |  |  |  |  |  |
| <30.0 | 94 | 8.8 (0 – 25) | 1.00 (0.86 – 1.15) | 0.90 (0.81 – 1.01) | 0.89 (0.80 – 0.99) |
| 30.0–49.9 | 590 | 9.0 (0 – 27) | 1.02 (0.95 -1.09) | 0.97 (0.92 – 1.03) | 0.97 (0.92 – 1.03) |
| 50.0–74.9 | 714 | 8.8 (0 – 28) | 1.00 (reference) | 1.00 (reference) | 1.00 (reference) |
| ≥75.0 | 207 | 8.3 (0 – 26) | 0.95 (0.85 – 1.05) | 0.91 (0.85 – 0.99) | 0.91 (0.84 – 0.98) |
| P for trend |  |  | 0.31 | 0.94 | 0.91 |
|  |  |  |  |  |  |
| Continuous^c^ | 1605 | 8.8 (0 – 28) | 1.03 (0.98 – 1.07) | 1.01 (0.98 – 1.05) | 1.01 (0.98 – 1.05) |

25(OH)D: 25-hydroxyvitamin D; 95% CI: 95% confidence interval; n: number of participants

^a^ Model 1 adjusted for age, sex, body mass index, occupation, marital status, smoking status in packyears, alcohol consumption, physical activity and diabetes.

^b^ Model 2 adjusted for osteoporosis, milk intake, sugary soft drink intake, smokeless tobacco use and depressive symptoms in addition to model 1.

^c^ per 25 nmol/L decrease in serum 25(OH)D.

**Supplementary table 4**. The association between seasonal-standardized serum 25(OH)D level and periodontal grades (n=1422)

| Serum 25(OH)D (nmol/L) |  | Ratio of means (95% CI) | | |
| --- | --- | --- | --- | --- |
|  | n | Crude model | Model 1^a^ | Model 2^b^ |
| Categorical |  |  |  |  |
| <30.0 | 75 | 1.03 (0.87 – 1.23) | 1.03 (0.86 – 1.22) | 1.02 (0.85 – 1.22) |
| 30.0–49.9 | 507 | 1.00 (0.92 – 1.09) | 1.00 (0.92 – 1.09) | 1.00 (0.92 – 1.09) |
| 50.0–74.9 | 653 | 1.00 (reference) | 1.00 (reference) | 1.00 (reference) |
| ≥75.0 | 187 | 1.02 (0.91 – 1.15) | 1.02 (0.90 – 1.15) | 1.02 (0.90 – 1.15) |
| P for trend |  | 0.97 | 0.94 | 0.94 |
|  |  |  |  |  |
| Continuous^c^ | 1422 | 0.99 (0.95 – 1.04) | 0.99 (0.94 – 1.04) | 0.99 (0.94 – 1.05) |

25(OH)D: 25-hydroxyvitamin D; 95% CI: 95% confidence interval; n: number of participants

^a^ Model 1 adjusted for age, sex, body mass index, occupation, marital status, smoking status in packyears, alcohol consumption, physical activity and diabetes.

^b^ Model 2 adjusted for osteoporosis, milk intake, sugary soft drink intake, smokeless tobacco use and depressive symptoms in addition to model 1.

^c^ per 25 nmol/L decrease in serum 25(OH)D.
